# How a menu of adherence support strategies facilitated high adherence to HIV prevention products among adolescent girls and young women in sub‐Saharan Africa: a mixed methods analysis

**DOI:** 10.1002/jia2.26189

**Published:** 2023-11-07

**Authors:** Sarah T. Roberts, Noah Mancuso, Kristin Williams, Hadijah Kalule Nabunya, Hlengiwe Mposula, Caroline Mugocha, Priscilla Mvinjelwa, Morgan Garcia, Daniel W. Szydlo, Lydia Soto‐Torres, Kenneth Ngure, Sybil Hosek

**Affiliations:** ^1^ Women's Global Health Imperative, RTI International Berkeley California USA; ^2^ Women's Global Health Imperative, RTI International Atlanta Georgia USA; ^3^ Applied Public Health Research Center RTI International Research Triangle Park North Carolina USA; ^4^ Makerere University‐Johns Hopkins University Research Collaboration Kampala Uganda; ^5^ Wits Reproductive Health and HIV Institute Johannesburg South Africa; ^6^ University of Zimbabwe Clinical Trials Research Centre Harare Zimbabwe; ^7^ Desmond Tutu HIV Centre Cape Town South Africa; ^8^ FHI 360 Durham North Carolina USA; ^9^ Statistical Center for HIV/AIDS Research and Prevention Fred Hutchinson Cancer Center Seattle Washington USA; ^10^ Division of AIDS National Institute of Allergy and Infectious Diseases, National Institutes of Health Bethesda Maryland USA; ^11^ School of Public Health Jomo Kenyatta University of Agriculture and Technology Nairobi Kenya; ^12^ Department of Global Health University of Washington Seattle Washington USA; ^13^ Center for Dissemination and Implementation Science University of Illinois Chicago Chicago Illinois USA

**Keywords:** adolescents, female, HIV, pre‐exposure prophylaxis, medication adherence, sub‐Saharan Africa, psychosocial intervention

## Abstract

**Introduction:**

Effective use of pre‐exposure prophylaxis (PrEP) has been low among adolescent girls and young women (AGYW) in sub‐Saharan Africa. The MTN‐034/REACH trial offered AGYW a menu of adherence support strategies and achieved high adherence to both daily oral PrEP and the monthly dapivirine vaginal ring. Understanding how these strategies promoted product use could inform the design of adherence support systems in programmatic settings.

**Methods:**

REACH was a randomized crossover trial evaluating the safety of and adherence to the ring and oral PrEP among 247 HIV‐negative AGYW (ages 16–21) in South Africa, Uganda and Zimbabwe from January 2019 to September 2021 (NCT03593655). Adherence support included monthly counselling sessions with drug‐level feedback (DLF) plus optional daily short message service (SMS) reminders, weekly phone or SMS check‐ins, peer support clubs, “peer buddies” and additional counselling. Counsellors documented adherence support choices and counselling content on standardized forms. Through focus groups, serial in‐depth interviews (IDIs) and single IDIs (*n* = 119 total), we explored participants’ experiences with adherence support and how it encouraged product use.

**Results:**

Participants received counselling at nearly all visits. DLF was provided at 54.3% of sessions and, across sites, 49%–68% received results showing high adherence for oral PrEP, and 73%–89% for the ring. The most popular support strategies were in‐person clubs and weekly calls, followed by online clubs, additional counselling and SMS. Preferences differed across sites but were similar for both products. Qualitative results demonstrated that the REACH strategies supported adherence by providing information about HIV and PrEP, continually motivating participants, and supporting the development of behavioural skills and self‐efficacy, aligning with the Information, Motivation, and Behavioural Skills (IMB) model. Effectiveness was supported by three foundational pillars: strong interpersonal relationships with counsellors; ongoing, easily accessible support and resources; and establishing trust in the counsellors and study products through counsellor relationships, peer‐to‐peer exchange and DLF.

**Conclusions:**

Implementation programmes could support effective PrEP use by offering a small menu of counsellor‐ and peer‐based support options that are youth‐friendly and developmentally appropriate. The same menu options can support both ring and oral PrEP users, though content should be tailored to the individual products.

## 1 INTRODUCTION

Adolescent girls and young women (AGYW) in sub‐Saharan Africa (SSA) account for roughly 25% of new HIV acquisitions while comprising only 10% of the population [1]. Daily oral pre‐exposure prophylaxis (“oral PrEP”) is an effective HIV prevention method [2], and the monthly dapivirine vaginal ring (“ring”) is safe and efficacious and is now recommended by the World Health Organization (WHO) for HIV prevention among cisgender women at substantial risk of HIV [3]. However, low adherence significantly reduces the effectiveness of both products in preventing HIV acquisition [2, 4, 5, 6]. Oral PrEP adherence has been low among AGYW across multiple studies in the region, and persistence in programmatic settings has averaged 2 months or less [7, 8, 9, 10, 11, 12, 13]. Young women have also had challenges with ring use, with significantly lower adherence than their adult counterparts in phase III trials [4, 14, 15, 16].

Barriers to oral PrEP use among cisgender AGYW exist across socio‐ecological levels, including low‐risk perception, daily pill burden and side effects; challenges with disclosure to and support from key influencers; limited community awareness and stigma; and clinic accessibility, stock‐outs and negative provider attitudes [6, 17, 18, 19, 20]. Facilitators to AGYW's oral PrEP use include youth‐friendly clinics, real‐time adherence feedback, social support and convenient, accessible supply [7, 18, 20, 21, 22]. There are limited data on ring use among adolescent girls, but barriers reported by adult women include fear of involuntary disclosure to partners during sex, side effects and perceived fertility concerns [22, 23]. To maximize the prevention impact of PrEP products among AGYW, interventions must address these adherence challenges. Though several PrEP adherence support interventions have been evaluated among AGYW in randomized trials, none have proven effective to date [9, 10, 13, 24, 25, 26].

In the MTN‐034 *Reversing the Epidemic in Africa with Choices in HIV Prevention* (REACH) trial, AGYW in SSA used both oral PrEP and the ring and were provided with counselling, drug‐level feedback (DLF) and the option to choose from a menu of additional strategies to facilitate effective product use. REACH participants had higher adherence to both products than AGYW in prior studies and demonstration projects [9, 10, 11, 12, 13, 15, 27, 28, 29, 30, 31]. Thus, this paper aims to describe the adherence support programme and investigate how it supported oral PrEP and ring use among study participants. Understanding how these strategies promoted product use could inform the design of future adherence support systems for PrEP delivery programmes with more limited resources.

## 2 METHODS

### 2.1 Study design and participants

REACH was a phase 2a, randomized, open‐label, crossover study conducted at four research sites in Cape Town and Johannesburg, South Africa; Kampala, Uganda; and Harare, Zimbabwe from January 2019 to September 2021. The primary objectives were to collect safety and adherence data for the ring and oral PrEP among AGYW and to understand a preference for the two products. The trial enrolled 247 HIV‐negative participants ages 16–21 years assigned female at birth. Participants were randomized 1:1 to use the ring or oral PrEP for one 6‐month period, then switched to the other product for a second 6‐month period.  In the third and final 6‐month period, participants could choose to use the ring, oral PrEP or neither product, and could switch products at any time. Detailed methods, participant characteristics and primary findings have been previously reported [27, 32, 33, 34].

### 2.2 Adherence support

**Figure 1:**
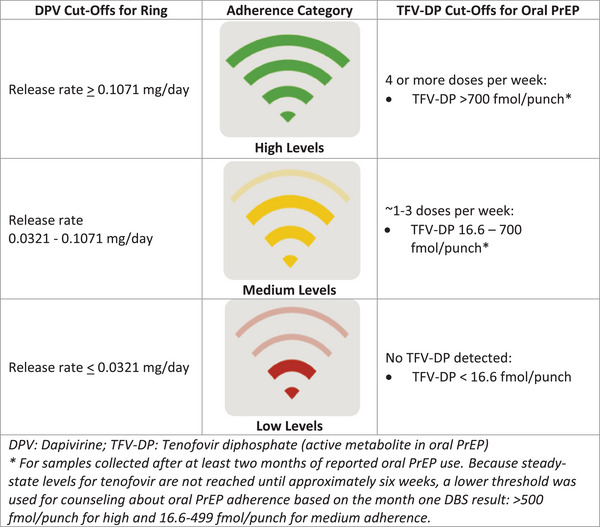
Wi‐Fi scheme to tailor drug‐level feedback messaging to study participants.

Participants received product adherence counselling from experienced counsellors at each monthly visit. Counsellors completed a 2‐day training and conducted up to 10 mock counselling sessions before seeing participants. Counselling used a client‐centred approach to frame discussions around empowerment and choice. Cognitive behavioural strategies were incorporated to address adherence barriers [35, 36, 37, 38, 39]. DLF was provided during counselling sessions using a Wi‐Fi schema (Figure 1) to initiate conversations about additional support [10]. DLF for the ring was based on residual dapivirine levels in used rings returned to the study clinics, and DLF for oral PrEP was based on intracellular tenofovir concentrations in dried blood spots (DBS) [4, 40, 41, 42]. Both measures reflected cumulative adherence over approximately 1 month. Green DLF results reflected levels of adherence associated with reduced risk of HIV in previous trials for both products [4, 28, 42, 43, 44]. Participants were instructed to return all rings, and DBS samples were collected at every follow‐up visit during oral PrEP use. Results were provided at the next adherence counselling session after they were returned from the lab, a median of 41.5 days after sample collection for oral PrEP and 56 days for the ring. Because results were sometimes delayed, multiple DLF results could be provided at the same visit.

At each visit, participants were invited to choose from a menu of additional adherence support strategies, including daily two‐way short message service (SMS) reminders, weekly two‐way SMS check‐ins, weekly phone calls, peer buddies, in‐person support clubs (all sites except Johannesburg), online support clubs, extra counselling sessions or other options of the participant's design. Support clubs (both in‐person and online) were facilitated by a site staff member to allow participants to regularly discuss the study, experiences with the pills and ring, and other issues going on in their lives. Participants could select multiple options and change options at any time. A pill‐carrying case was also offered to all participants upon oral PrEP initiation and replaced if needed. These additional support strategies, coupled with the monthly counselling, formed participants’ individualized adherence support plans. Each study site also endeavoured to provide youth‐friendly services that were *accessible, acceptable, equitable, appropriate and effective*, according to WHO standards [45, 46].

In March 2020, to comply with social distancing guidelines during the COVID pandemic, in‐person support clubs were paused and additional counselling sessions were offered by phone instead of in‐person (Table S1).

### 2.3 Present study

This mixed methods analysis used a concurrent triangulation design to better understand the REACH adherence support programme [47]. We used quantitative data to describe the dosing of the adherence support strategies, and qualitative data to understand how the strategies worked. Both data sources were combined for interpretation of results and to make recommendations.

### 2.4 Quantitative measures and analysis

Counsellors documented adherence counselling content, DLF provision and participants’ choices for additional support strategies on a standardized form. When menu options were restricted during COVID, counsellors documented which options participants would prefer, even if they were not available. During analysis, we determined which strategies were actually “used” by deleting options not available at the visit date (assuming that participants used all strategies they selected and were available to them). Descriptive statistics were calculated with Stata v.17, stratified by PrEP product and study site.

### 2.5 Qualitative measures and analysis

#### 2.5.1 Data collection

A subset of participants (*n* = 119/247; 48.2%) across all sites took part in serial in‐depth interviews (SIDIs), single in‐depth interviews (IDIs) and focus group discussions (FGDs). Details of each activity are provided in Table 1. Interviews and FGDs were conducted in local languages by trained, local social scientists in a private setting, using semi‐structured guides. Based on the Mensch Acceptability Model [48] and Psychological Empowerment Framework [49], topics included experiences with study participation and product use (including the adherence support strategies), and product acceptability, preferences and choice. Discussions were audio‐recorded, transcribed and translated into English, with a thorough quality control review before finalization.

**Table 1:** Qualitative data collection activities

Activity	Sample size	Participant selection	Timing	Average duration
Serial in‐depth interviews (SIDIs): 3 per participant	24 participants	Random. From each site, one participant was randomly selected from each of six strata defined by age group (16–17, 18–19 and 20–21) and product assignment for the first study period and invited to participate. If they refused, the next randomly assigned participant was invited.	Two months into each product use period	60 minutes
Single in‐depth interviews (IDIs)	38 participants^a^	Purposive. Sample includes all participants who chose neither product at the beginning of the third product use period and “special cases” representing unexpected or interesting experiences and behaviours relevant to the study endpoints, such as social harms, seroconversion or advocacy. Special cases were nominated by site staff and approved by the qualitative data management team.	Upon identification	60 minutes
Focus group discussions (FGDs)	16 FGDs 62 participants^a^ (3–6 participants per FGD)	Purposive. Sought to include a range of ages, product choices and adherence levels based on DLF results.	In the third product use period, between month 15 and study exit	90 minutes

^a^ Five participants took part in both single IDIs and FGDs.

#### 2.5.2 Analysis

A codebook was developed by team members from the qualitative data management centre (QDMC) at RTI International, the protocol team and research sites through an iterative inductive and deductive process. Initial codes were deduced from the research objectives, the conceptual frameworks [48, 49] and codebooks from previous similar studies [50, 51, 52], and induced from themes emerging in debrief reports [53]. Revisions occurred after further review of transcripts and debrief reports; a workshop with site staff, QDMC and study leadership; and test coding of transcripts from one FGD, one IDI and one set of SIDIs. Transcripts were then coded using Dedoose software v9.0.17 by six analysts from the QDMC and one research site. Senior social scientists from each site reviewed a selection of coded transcripts, and the QDMC assessed inter‐coder consistency with the Dedoose training tool and weekly transcript review. Discrepancies were discussed and resolved through consensus.

For this analysis, the lead author and two co‐authors reviewed all excerpts coded with the *Adherence Support Intervention*s or *Drug‐Level Feedback* codes, stratified into nine code reports based on co‐coding with *Self‐Efficacy/Resilience; Motivation; Barriers; Facilitators and Strategies; Initiation/Early Use, Execution/Compliance or Discontinuation; Choice; Social Harm/Benefit;* or none of the above. (See Table S2 for code definitions.) Summary memos were created for each code report, then refined after biweekly meetings with counsellors from each site, the analysts, and the first and senior authors. Although we initially selected the Psychological Empowerment Theory to investigate how the support strategies encouraged PrEP use, themes arising in the summary memos aligned more closely to the constructs of the Information, Motivation, and Behavioural Skills (IMB) model [49, 54], and that model was adopted for further analysis. The domains in the IMB model were defined as: information (didactic knowledge about product use); motivation (intentions and attitudes towards product use) and behavioural skills (self‐efficacy and execution of strategies for product use).

### 2.6 Ethics statement

The study protocol was approved by the Institutional Review Board or Ethics Committee at each site (Table S3). The study was overseen by the regulatory infrastructure of the Division of AIDS and the MTN. All adult participants provided written informed consent for study participation, including interviews and FGDs. Following local regulations, written assent and parent/guardian permission were obtained for participants below the legal age of consent.

## 3 RESULTS

### 3.1 Baseline characteristics

The median age among the 247 participants was 18 years (interquartile range [IQR] 17–19). Most were unmarried (87%), had a primary sex partner (89%), had attended secondary school (86%) and lived with a parent (70%). Four in ten were “very” or “somewhat” worried about acquiring HIV in the next year. The majority had mobile phones (85%), of whom 25% shared their phone with someone else. The qualitative subsample was comparable to the overall study sample on socio‐demographic measures (data not shown).

### 3.2 Counselling frequency and content

All participants had adherence counselling at enrolment. The mean number of follow‐up sessions over 18 months was 16.4 (standard deviation [SD]: 3.6). Counsellors reported discussing adherence goal setting, reminder strategies, barriers to adherence, communication skills and product storage at >90% of sessions (Figure S1). Reported discussion of other topics varied widely by site, but were similar during periods of oral PrEP use and ring use.

**Figure 2:**
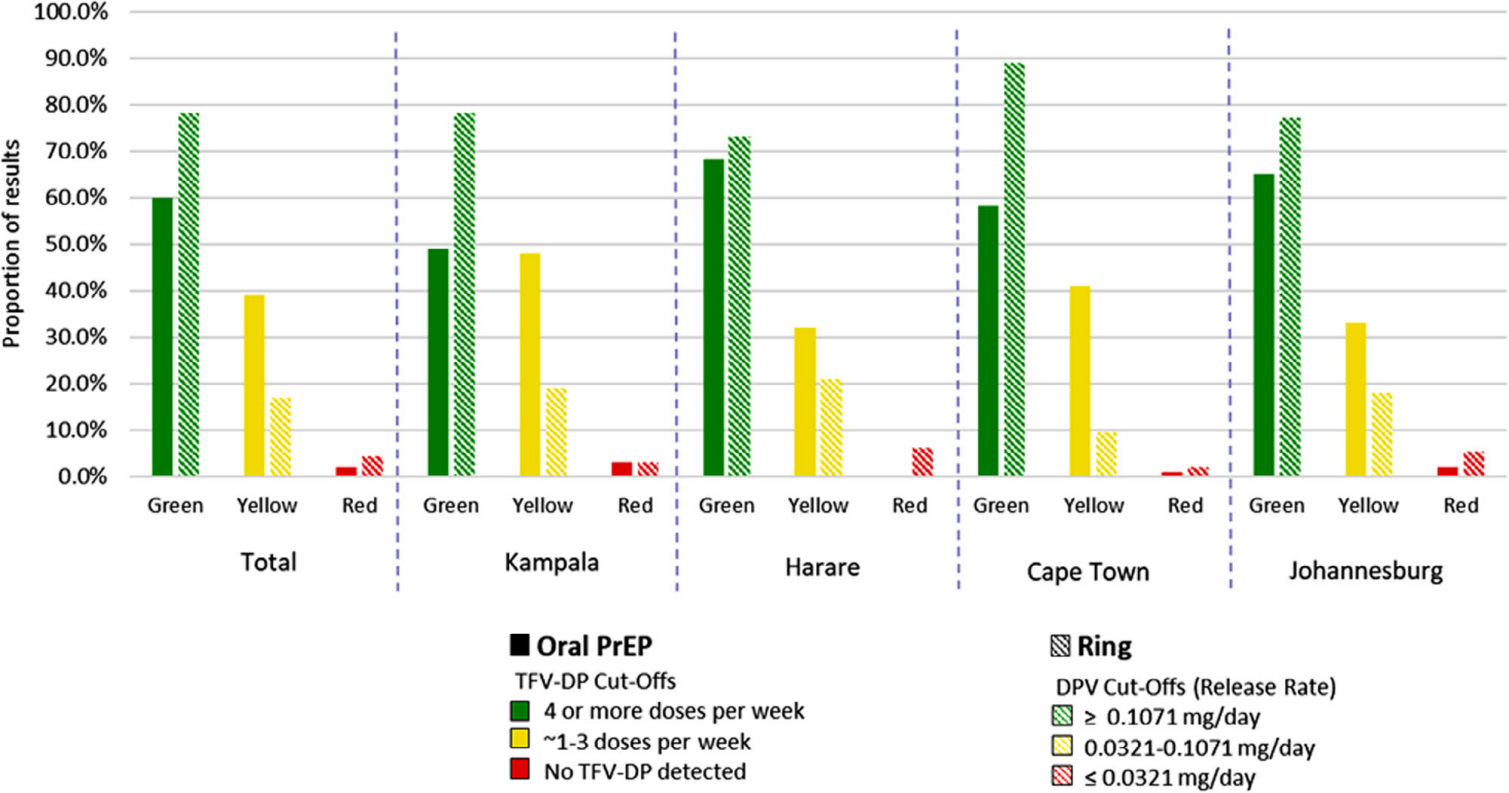
Drug‐level feedback results by product and site. A total of 2985 results were returned to participants (1260 for oral PrEP adherence and 1725 for ring adherence), including 854 at the Kampala site (361 oral PrEP, 493 ring); 854 at the Harare site (329 oral PrEP, 416 ring); 516 at the Cape Town site (228 oral PrEP; 288 ring); and 870 at the Johannesburg site (342 oral PrEP, 528 ring).

DLF was available at 2031 monthly visits (55%), with >1 result available at 29% (*n* = 589). Participants received a median of 13 DLF results during the study (IQR: 10–15). The proportion of visits with DLF results available declined notably during the initial lockdowns of the COVID‐19 pandemic (Figure S2). Across sites, 49%–68% of returned DLF results were “green” for oral PrEP and 73%–89% for the ring (Figure 2). Participants received green results at an average of 56% (SD: 39%) of their sessions for oral PrEP and 78% (SD: 26%) of their sessions for the ring.

### 3.3 Uptake of additional adherence support strategies

**Figure 3:**
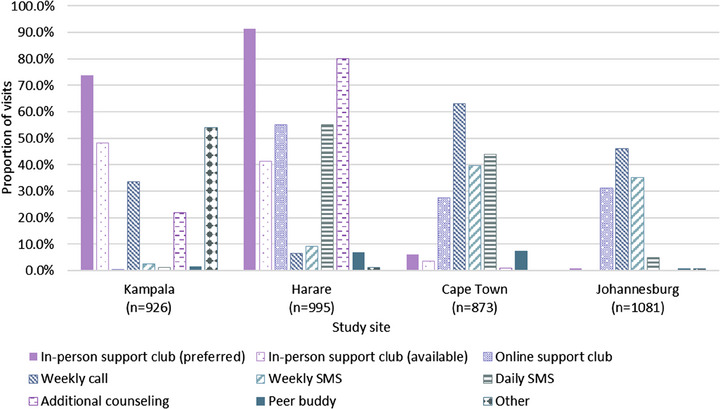
Additional adherence strategies selected at each visit, by study site. *Notes*: Because in‐person support clubs were not available at times due to COVID restrictions, we show the proportion of visits at which this option was preferred (first bar) and used (i.e. both and available) (second bar) separately. This distinction does not apply to the other options because they were available consistently throughout the study. Although no in‐person support clubs were offered in Johannesburg, they were documented as selected or preferred by eight participants at 10 visits. “Other” methods selected were predominantly phone calls at less‐than‐weekly frequencies.

The most popular adherence support strategies were in‐person support clubs, selected at 42.7% of sessions (though only used at 22.9% due to COVID restrictions), and weekly calls (36.8%), followed by online support clubs, additional counselling, daily SMS and weekly SMS. There were substantial differences across sites (Figure 3). Daily SMS was selected more often during oral PrEP use (30.0%) than ring use (22.2%), but the selection of other strategies was similar (Table S4).

Participants chose a median of two additional strategies per visit (IQR 1–2), ranging from 1 (IQR 1–2) in Johannesburg to 3 (IQR 2–3) in Harare, with slightly more strategies selected during oral PrEP use (median 2, IQR 1–3) than ring use (median 2, IQR 1–2). Table 2, Panel 1, summarizes the selection of peer support methods (support clubs and peer buddies) and counsellor methods (additional counselling, calls and SMS) at each study visit. Participants from Kampala and Harare overwhelmingly preferred a combination of peer‐based and counsellor‐based methods (selected at 73.0% and 86.7% of visits, respectively), though actual use of the combined approaches was less frequent due to COVID restrictions on in‐person clubs. Participants from South Africa predominantly preferred and used counsellor‐based support methods (61.1% in Cape Town and 66.3% in Johannesburg). There were no notable differences in combinations chosen by the study product. Results were similar when aggregated at the participant level by describing which combination of strategies they chose at ≥50% of study visits (Table 2, Panel 2).

**Table 2:** Types of additional adherence support strategies selected by participants by site

Additional support sources	All sites		Kampala		Harare		Cape Town		Johannesburg	
	*n*	%	*n*	%	*n*	%	*n*	%	*n*	%
***Panel 1. Visit‐level data***										
Number of visits	3875	100.0	926	100.0	995	100.0	873	100.0	1081	100.0
*1A: Preferred (selected among all options)*										
Peer only	407	10.5	24	2.6	91	9.2	53	6.1	239	22.1
Counsellor only	1513	39.1	225	24.3	38	3.8	533	61.1	717	66.3
Peer and counsellor	1912	49.3	676	73.0	863	86.7	258	29.6	115	10.6
Neither	43	1.1	1	0.1	3	0.3	29	3.3	10	0.9
*1B: Used (selected among available options)*										
Peer only	392	10.1	13	1.4	91	9.2	52	6.0	236	21.8
Counsellor only	1915	49.4	450	48.6	200	20.1	543	62.2	722	66.8
Peer and counsellor	1510	39.0	451	48.7	701	70.5	248	28.4	110	10.2
Neither	58	1.5	12	1.3	3	0.3	30	3.4	13	1.2
***Panel 2. Participant (ppt)‐level data***										
Number of ppts	247	100.0	60	100.0	60	100.0	60	100.0	67	100.0
*2A: Preferred (selected among all options) at ≥50% of visits*										
Peer only	15	6.5	0	0.0	1	1.7	2	3.3	13	19.4
Counsellor only	90	36.4	9	15.0	0	0.0	37	61.7	44	65.7
Peer and counsellor	129	52.2	51	85.0	59	98.3	15	25.0	4	6.0
Neither^a^	12	4.9	0	10.0	0	0.0	6	10.0	6	9.0
*2B: Used (selected among available options) at ≥50% of visits*										
Peer only	16	6.5	0	0.0	1	1.7	2	3.3	13	19.4
Counsellor only	114	46.2	23	38.3	10	16.7	37	61.7	44	65.7
Peer and counsellor	102	41.3	34	56.7	49	81.7	15	25.0	4	6.0
Neither^a^	15	6.1	3	5.0	0	0.0	6	10.0	6	9.0

*Note*: Peer‐based strategies include in‐person support clubs, online support clubs and peer buddies. Counsellor‐based strategies include additional counselling, weekly phone calls and daily or weekly SMS. Panel 1 shows visit‐level data. Panel 2 categorizes preference and use for each participant according to which combination of strategies they chose at ≥50% of study visits.

^a^ In the participant‐level analysis, 12 participants (six in Cape Town and six in Johannesburg) did not prefer or use any combination >50% of the time. An additional three participants in Kampala did not use any combination >50% of the time.

### 3.4 Qualitative results: overview

**Figure 4:**
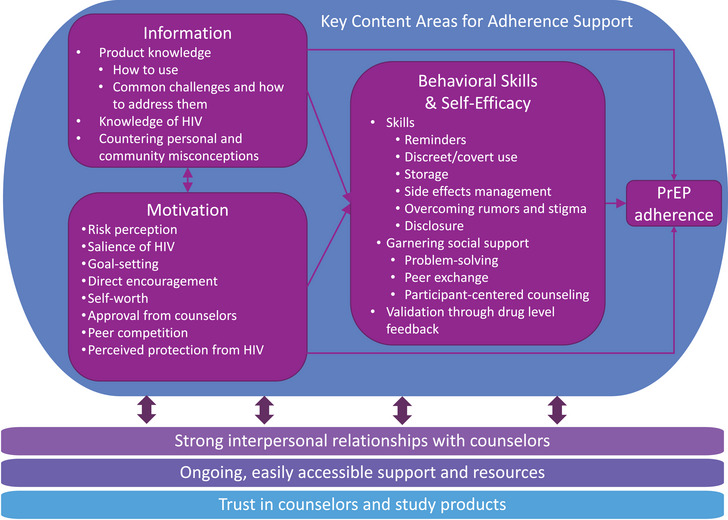
Conceptual model of mechanisms of adherence support in the REACH study.

Counselling, DLF and additional menu options supported adherence through the constructs of the IMB model [54]. These pathways’ effectiveness was supported by three foundational pillars: strong interpersonal relationships with counsellors; ongoing, easily accessible support and resources; and establishing trust in the counsellors and study products (Figure 4). Below, we describe these constructs and underlying pillars in turn.

### 3.5 Information

Participants valued the thorough knowledge about HIV and the study products that they gained through counselling and peer support clubs. Understanding how HIV is transmitted and how the products work increased AGYW's trust that the products could truly prevent HIV, and thus their motivation to use them.

*The benefit for me was knowledge because I didn't know…Like that it can kill a person when she does not take treatment and all that. So …REACH came with the knowledge that you are able to protect yourself and not have it, not get it. (FGD, Cape Town, age 18)*

Participants also gained knowledge about managing product use in routine and unanticipated situations, such as missing their scheduled dosing time, reinserting the ring after removal, feeling unwell or suspecting pregnancy, and this knowledge built self‐efficacy to adhere. Information about adverse events relieved participants’ product safety fears, which often stemmed from community rumours. Though this information initially came from counsellors, many AGYW were convinced of its truth after support clubs. Hearing peers’ experiences reassured participants that their side effects were normal or expected, and they were motivated by seeing others’ side effects subside or be successfully managed:

*And also the adherence support group, where you help each other by sharing your problems, others will encourage you not to be afraid. … you may be thinking of leaving the pills because of certain reasons but you can meet with someone who would have experienced the same challenge and it stopped and they encourage you not to quit. So, you will see that you will continue taking the pills and not quit. (Serial IDI, Harare, age 18)*

The support clubs also helped participants ask questions they initially felt embarrassed about, further increasing knowledge and supporting adherence.

*For example, the rumours spread about the pills by the community…I would keep quiet about it and this affected my adherence. So when I heard participants talk, I decided to talk so that I am helped or even know the fact whether it is true that they affect the liver, causes cancer, HIV. So when I opened up I learnt the truth and was helped. (Serial IDI, Kampala, age 18)*

Additionally, information helped participants explain the products to people outside the study. During disclosure, this enabled them to project confidence in their choice to use a PrEP product. It also increased their ability to confront or disregard rumours or misinformation and stay motivated despite what others said.

*…People have their own views and opinions. Like, “Aren't you scared, what if it gives you a reaction, will they help you when it reacts, what if it doesn't agree with you as you know people differ?” I was like well you know I was counselled at the clinic so they gave me a full understanding…’’ (Serial IDI, Johannesburg, age 19)*

### 3.6 Motivation

The support strategies motivated adherence by increasing risk perception and salience of HIV, helping participants set and strive for adherence goals, providing direct encouragement to build confidence, and making participants feel supported and invested in. Examples are illustrated by the quotes below:

*Risk perception: I thought that I could not get HIV. I was saying, after all I have one male partner … Then the study staff told me, “Do you know where your male partner is all the time? Do you get to know who he is with all the time?” and I said no. Then I became serious with taking tablets. (Single IDI, Uganda, Age 16)*

*Direct encouragement: … Then [the counsellor] won't be harsh on me “why don't you take your pills whatsoever.” She will encourage me to take my pills all the time because it's for my health, yes. (Serial IDI, Cape Town, age 18)*

*Support: I'm still way more encouraged to take [the study products] because of the adherence group that we also have with other girls, they check up on us if everything is still going well, so you never feel discouraged with using it because there is always someone there to help if ever there is a problem. (Serial IDI, Johannesburg, age 19)*

DLF played an important role in motivating product use, and some participants admitted that they would not use the study products without it. Most often, this motivation was driven by the desire to avoid negative reactions from counsellors, whose approval AGYW were eager to gain. Many reported using the products so that they would not have to admit to poor adherence.

*Chloe: [Drug level feedback] helped me because when I first started using tablets [oral PrEP] I used to skip. Then, I didn't know that there was drug level feedback. So, sometimes I would tell aunt that I took them every day, so [All Laugh]‐ so, that is when I realized that these people [staff] know that this person is not using tablets. So, I ended up taking my tablets well*.

*Lucy: With the Wi‐Fi there is no lying. (FGD, Harare, ages 17 and 18)*

DLF also created friendly competition among peers, motivating them to match or beat each other's results, and some study sites encouraged this by rewarding those with consistently green results with treats like cake or lipstick, or with invitations to speak as role models during support clubs. For a smaller number of AGYW, receipt of red or yellow DLF results motivated higher adherence to get more protection for its own sake. This reflected widespread trust among most participants that the results accurately reflected their level of HIV protection, which stemmed from a clear understanding of how to interpret the results and overwhelming agreement that the results accurately represented their adherence behaviour.

In rare cases, however, receiving yellow or red results led to high levels of stress, anxiety or shame, and became a barrier to using PrEP products or accessing additional support. One AGYW quit the support club because she felt she had nothing to offer if she was not getting green results, and another chose to use neither product during period 3 because she did not believe either would protect her.

### 3.7 Behavioural skills and self‐efficacy

Participants reported learning behavioural skills to overcome adherence challenges during counselling sessions and support clubs, and these skills were supported by phone and SMS contacts and the pill‐carrying case. Frequently mentioned problem‐solving approaches included: reminder systems for daily pills, ring replacement or study visits; discreet use to avoid unwanted disclosure; managing product storage at home and while travelling; practices to reduce or treat side effects; ignoring or dispelling rumours and stigma; and strategies to disclose product use and gain adherence support from people outside the study. Many participants successfully addressed their product use challenges by applying these skills and mobilizing study resources. However, a few reported ongoing challenges with food, side effects or other factors; not receiving adequate counselling or support on disclosure; not receiving the texts or phone calls they signed up for; or that their attempts to gain external support were unsuccessful.

Participants reported that counsellors helped them identify and prioritize their own adherence challenges and determine the best problem‐solving strategies. This approach increased AGYW's perceived self‐efficacy to address these challenges and imparted a sense that it was their responsibility to take or leave the counsellors’ advice.

*[The counsellor] asked me what is going to challenge me not to use the pills. I told her that I go to school and they said I need to find a way that will help to me to take the pills. Then I decided to take them at night always. So they helped me to identify the problem. Then I came with the solution on my own on what to do. (FGD, Cape Town, age 16)*

Problem‐solving usually occurred in this manner and through brainstorming with peers, but counsellors also provided more direct support at times, for example by meeting with family members or partners to facilitate product use disclosure or dispel rumours, or by referring participants to clinicians to manage side effects. The phone calls and SMS contacts facilitated fast access to this support, helping AGYW address side effects quickly instead of having to wait until their next scheduled visit. Participants’ sense of self‐efficacy was strengthened by seeing these approaches succeed for themselves and their peers.

Receiving green DLF results built additional self‐efficacy by validating participants’ adherence efforts. Although AGYW could usually predict their results from their actual product use, many relied on the DLF to gauge whether they were truly protected, and felt stress and uncertainty about their protection levels when results were delayed. Conversely, receipt of yellow or red DLF was critical in helping participants access counselling on behavioural skills by opening the door to honest discussions of adherence challenges:

*Even if I lie to her [the counsellor] the results would come and tell the truth and she would be like “you lied” and I would say “I know” let me fix it… When the results come back she will be like “let's talk about it” and then we talk about it. (FGD, Johannesburg, age 18)*

### 3.8 Foundational pillars

The findings above highlight that three foundational pillars supported these pathways to adherence and were essential to their effectiveness:

1. *Strong interpersonal relationships with counsellors*. Counsellors were overwhelmingly seen as friendly, approachable and non‐judgemental. Their responses to yellow or red DLF were reported to be positive and solution‐focused, and only one participant reported a harsh reaction that made her want to quit the study. AGYW felt the counsellors were truly invested in their success with the products and in their lives more broadly, which was a new experience for many. The resulting discussions often relieved stress or increased feelings of self‐worth, increasing capacity and motivation for adherence.
*Sometimes we come from home with problems but the moment we enter the counselling room… They start saying, “Aah my niece, is there anything that is troubling you?…” When you share your problems, whether they are related to the study or not, they will counsel you. When you leave there you will be happy, forgetting that you had a burden. (FGD, Harare, age 17)*

In combination with DLF, these strong relationships also motivated participants to adhere to avoid disappointing counsellors who had invested in them. Furthermore, they made AGYW more willing to open up to counsellors, ask questions and share challenges, which allowed counsellors to help them more effectively.

2. *Ongoing, easily accessible support and resources*. The adherence support system provided multiple ways for participants to frequently engage with counsellors and peers, and frequent DLF results provided ongoing validation and feedback. These touchpoints made it easy to access resources, such as clinical care, referrals and additional counselling for themselves and their partners or family members, building motivation and self‐efficacy. This accessibility was particularly valued by participants at the South African sites.
*What I like is that … when you need help, you must always call and all that. So help is always available. (FGD, Cape Town, age 16)*
3. *Trust in the counsellors and the study products*. At study enrolment, most participants had limited knowledge about HIV and PrEP. Many were sceptical about the safety and efficacy of products, their ability to use them and the intentions of the study staff—an attitude that was reinforced by rumours circulating in the community. However, the open, non‐judgemental relationships increased their trust in the information and advice the counsellors provided. The alignment of participant's experiences with DLF results assured them that the results were accurate, the counselling activities were worthwhile and the products truly prevented HIV, a belief that was reinforced by repeated negative HIV test results. Finally, interactions with peers created important opportunities to observe the outcomes of the counselling and product use:
*You might have been given PrEP and you vomited and you were like “I have vomited, I think others are not vomiting” so, when they call you for a [support club] and you share your challenge of vomiting then you hear another girl saying “I also experienced it” and another and another and then you get to know that the counsellor was not lying when she said “You might vomit, feel a little dizzy” and then I get to know “Well, it is true.” (Single IDI, Kampala, age 16)*

Over the course of the study, these factors coalesced to build trust and convince participants that they could successfully and safely use the products to achieve ongoing HIV prevention.

## 4 DISCUSSION

Through the REACH adherence support system, participants received frequent counselling and DLF and had high levels of engagement in optional support strategies. Three key findings emerged about how the support system worked. First, regardless of the strategies chosen, AGYW reported benefitting most through pathways aligned with the IMB model of behaviour change: information about HIV and PrEP products, motivation to continue product use, and skills and resources to build self‐efficacy to adhere. Second, for this approach to be successful, participants had to develop close relationships with counsellors, be able to engage frequently and easily with the support system to access additional help and resources, and gain trust in the products and counsellors. Third, although there was wide variation in the menu options selected within and across sites, similar support strategies were used for oral PrEP and the ring, and all sites had similar rates of high adherence.

Observational studies have supported the use of the IMB model to characterize barriers and facilitators of PrEP and antiretroviral therapy use among men who have sex with men [55, 56, 57, 58] and people who use drugs [59]. This study expands evidence for the model to PrEP adherence among a new population, AGYW in SSA, and is one of the first to demonstrate IMB constructs as mechanisms of action in an adherence support intervention [60]. Following the approach developed by Amico and colleagues [61], our model is “situated” to reflect the context of AGYW's PrEP decision‐making. For example, the information domain includes the knowledge to counter misconceptions about PrEP, the motivation domain includes self‐worth and the desire for counsellors’ approval, and the behavioural skills and self‐efficacy domain includes disclosure, garnering social support and overcoming rumours, all key challenges that participants had to navigate to successfully adhere. Importantly, the REACH support strategies went beyond counselling to provide additional resources—such as referrals, family and partner counselling, and clinical care—that helped address these contextual barriers.

The three foundational pillars of the REACH approach—strong interpersonal relationship, ongoing support and trust—emphasize the importance of providing youth‐friendly and developmentally appropriate adherence support [62]. Previous research has also identified the benefit of frequent, ongoing support and noted declining PrEP adherence with a switch from monthly to quarterly visits [63, 64, 65, 66]. A study in Kenya found that one‐way daily SMS reminders had no impact on oral PrEP adherence. However, REACH participants reported that daily or weekly calls or two‐way SMS made them feel that support was accessible and facilitated their adherence [9]. Reminders may need to be interactive so AGYW perceive a caring person on the other end of the line from whom they can get help. Adolescents often question authority as they develop new critical thinking capacities, and REACH participant's scepticism of the products and the counsellors may be a natural developmental phase [67, 68]. Strong relationships with providers, peer‐to‐peer exchange and validation by DLF helped REACH participants overcome scepticism and build trust, and motivated them to engage with the counselling content and commit to product use. Though many studies and national guidelines call for youth‐friendly services to provide these foundational pillars [69, 70, 71, 72, 73, 74, 75], they are rarely implemented with consistency or fidelity [76, 77, 78, 79, 80]. Our findings echo others’ in identifying youth‐friendly services as essential for success in PrEP delivery.

A substantial barrier to replicating the REACH adherence support system in programmatic settings is the provision of DLF, due to cost and logistical challenges. In the HPTN 082 study, which found no effect of DLF on PrEP adherence [10], DLF was provided at months 2 and 3 only, and the primary outcome was PrEP adherence at month 6. REACH participants received DLF throughout the study and reported that the repeated validation built self‐efficacy and motivated persistence. Our findings suggest that less frequent DLF may not provide adequate feedback to support adherence. Alternative, lower‐cost strategies are needed for programmatic settings to reinforce AGYW's adherence successes and facilitate honest reporting of adherence challenges.

In our quantitative analysis, no single combination of additional menu options worked best to support adherence, but the same options were preferred during the use of both products. These findings suggest that PrEP delivery programmes should offer a choice of adherence support methods, including counsellor‐ and peer‐based options, as well as in‐person and mHealth options, to suit individual preferences and circumstances. Evidence for improved adherence from other regions and health conditions similarly highlights the promise of multi‐modal interventions that include education, counselling, youth‐friendly services and mobile technology [81, 82, 83, 84]. The ability to offer the same options to users of different PrEP products may increase the feasibility of providing a menu of strategies to AGYW in programmatic settings; however, specific messages will still need to be tailored to barriers and facilitators for each product, as they were in REACH. Our findings cannot be extrapolated to products beyond daily oral PrEP and the ring. It will be important to explore what strategies support AGYW's use of injectable PrEP and other products as they become available in the future.

This study has several limitations. REACH participants were highly motivated, committing to 18 months of product use, and are not representative of the general population of AGYW in SSA. However, participants’ baseline characteristics [33], including high prevalence of sexually transmitted infections, depression and transactional sex, indicated vulnerability to HIV, suggesting that they represent a subset of AGYW who can benefit from support to achieve prevention‐effective adherence [85]. Additionally, REACH sites sought to ensure positive experiences for participants to enhance retention. Our evaluation focused on the adherence support programme and may have missed other efforts that supported product use. Finally, our self‐reported data may have been subject to social desirability bias. We selected IDI and FGD facilitators who were not involved in other study operations to reduce this bias.

## 5 CONCLUSIONS

The REACH adherence interventions facilitated PrEP use by providing information about HIV and PrEP, continually motivating participants and supporting them to develop behavioural skills and self‐efficacy. These strategies supported adherence because they were delivered in a youth‐friendly environment with caring, non‐judgemental counsellors; provided frequent opportunities to engage with counsellors and peers and to access additional support and resources; and built trust in products and staff. Retention of these core components in programmatic adaptations of the REACH strategies may help ensure their success in supporting oral PrEP and ring use among AGYW.

## AUTHORS’ CONTRIBUTIONS

STR and SH conceptualized the manuscript. STR, NM and KW analysed the data and SH, HNK, HM, CM and PM contributed to the interpretation of results. STR, MG, DWS, LS‐T, KN and SH were involved in the study design and overall study implementation. HNK, HM, CM and PM were involved with study implementation and data collection at the sites. STR and NM wrote the original manuscript draft. All co‐authors participated in revising the manuscript and approved the final version.

## COMPETING INTERESTS

The authors declare that they have no competing interests.

## FUNDING

This study was designed and implemented by the Microbicide Trials Network (MTN) funded by the National Institute of Allergy and Infectious Diseases through individual grants (UM1AI068633, UM1AI068615 and UM1AI106707), with co‐funding from the Eunice Kennedy Shriver National Institute of Child Health and Human Development and the National Institute of Mental Health, all components of the U.S. National Institutes of Health (NIH).

## DISCLAIMER

The content is solely the responsibility of the authors and does not necessarily represent the official views of the National Institutes of Health. The International Partnership for Microbicides provided the dapivirine vaginal rings for this study.

## Supporting information


**Table S1**: Changes to REACH adherence support activities due to the COVID‐19 pandemic.
**Table S2**: Definitions for codes used in analysis for this manuscript.
**Table S3**: Ethics committees approving the REACH trial.
**Table S4**: Additional adherence strategies selected at each visit, overall and by product type.
**Figure S1**. Topics discussed during counseling sessions.
**Figure S2**: Percent of visits with drug level feedback results available, by site and calendar month.

## Data Availability

The data that support the findings of this study are available from the corresponding author upon reasonable request.
