# Supplementary material for: How a menu of adherence support strategies facilitated high adherence to HIV prevention products among adolescent girls and young women in sub‐Saharan Africa: a mixed methods analysis
Source: J Int AIDS Soc. 2023 Nov 7;26(11):e26189. doi: 10.1002/jia2.26189 (PMC10630658; doi:10.1002/jia2.26189)

**Supplemental Information File 1: Supplemental Tables and Figures**

**Supplemental Table 1: Changes to REACH adherence support activities due to the COVID-19 pandemic.**

| **Site** | **Description of adherence support activity and change** | **Date that change was initiated** | **Date that normal activities resumed** |
| --- | --- | --- | --- |
| Kampala | Pause in-person adherence support clubs | 06 March 2020 | 14 August 2020* |
| Harare | Pause in-person adherence support groups | 20 March 2020 | Not resumed |
|  | Pause in-person counseling sessions; offer additional counseling sessions by phone in place of in-persons sessions. | 30 March 2020 | Not resumed |
| Cape Town | Pause in-person adherence support groups | 27 March 2020 | 17 May 2021 |
|  | Pause in-person counseling sessions; offer additional counseling sessions by phone in place of in-persons sessions. | 27 March 2020 | 19 June 2020 |
| Johannesburg** | Pause in additional in-person counseling support; offer additional counseling sessions by phone in place of in-persons sessions. | 27 March 2020 | 05 May 2020 |

* Resumed in small group format (2-3 ppts per session)

** Did not offer in-person support clubs.

**Supplemental Table 2: Definitions for codes used in analysis for this manuscript.**

| **Code** | **Definition** |
| --- | --- |
| Adherence Support Interventions (KC) | Discussion of any adherence support strategies, including counseling, adherence support meetings, SMS, phone calls, or product storage tools (e.g., keychains). Comments about drug level results should be coded DRUG LEVEL FEEDBACK. |
| Drug Level Feedback (KC) | Discussions around receiving drug level results i.e., their understandings of the level of protection scores (red, yellow, green), what their results were and whether they agree/disagree, and how the results affected product use. |
| Self-efficacy/ Resilience | Discussion on belief about one’s ability and capacity to accomplish a task or cope with environmental demands, e.g., belief in having the skills needed to achieve high adherence. Co-code with ADHERENCE SUPPORT INTERVENTION, DRUG LEVEL FEEDBACK, RING or PILL, when applicable. |
| Motivation for adherence | Any discussion of what motivates (or does not motivate) the participant to adhere to product use. Could include life goals/planning, encounters/exposure with people living with HIV either at home or in the study. Co-code with RISK or EFFICACY if applicable. |
| Social harm/benefit | Use to capture described benefits OR harm related to study participation or product use. Double code with other appropriate codes, e.g., VIOLENCE, DEVELOPMENTAL GROWTH, MONEY. |
| Choice (KC) | Decision-making, reflections on choice process for ring vs pills and factors in decision-making, including using nothing in period 3. Also include discussions about anticipated choice of product (e.g., what participant will potentially choose in period 3). |
| Initiation and early use (KC) | Any discussion on thoughts and feelings about product use leading up to start of product use or shortly after initiation (could also be applicable prior to switching products or re-starting product use after a period of non-use). Include comments on fearing/struggling to use the product in the early months of use. Co-Code with EXECUTION/COMPLIANCE when discussing non-adherence (e.g., missing/forgetting doses or ring removal) soon after initiation. |
| Execution/ compliance (KC) | Discussion of experiences using the study products or adhering to product use, including missing/forgetting doses, & ring removals, expulsion, or  slippages, and pauses or other noncompliant use of the products by ppt (e.g., cutting the pills in 2, skipping days or double dosing, not removing the ring after 1 month etc..). This includes unintended gaps of less than 1 month in product use due to external circumstance (COVID, travel, sickness, IPV, etc.. ). |
| Discontinuation (KC) | Discussion of stopping to use the ring or tablet permanently or for at least 1 month, or discussion of not stopping product use through the expected 6-month duration on product. Stopping can be due to any reason (voluntary, involuntary, or clinical reasons and includes end of study, pregnancy, HIV seroconversion, etc.). Brief pauses or temporary stops (less than 1 month) followed by restarting should be coded as “execution”. Clinic driven product holds (temporary or permanent) should always be coded discontinuation regardless of the length of the hold. Use EXECUTION/COMPLIANCE if there is no explicit discussion of a clinic-based hold and it’s unclear if the duration of the pause is more or less than 1 month. |
| Facilitators & Strategies (KC) | Any discussion of factors or strategies that help with product adherence. Include reminders, other adherence aids/strategies, and community/family social support (buddy system). Co-code with EXECUTION/COMPLIANCE when accompanied by discussion about how the facilitator/strategy actually resulted in good/improved compliance with product use. |
| Barriers (KC) | Any discussion of barriers/challenges (or lack of) related to product use, i.e., issues of discreetness/privacy, opposition from others, attitudes towards using a product when not sick/low risk perception, etc. Also include negative experiences that the participant does not identify explicitly as barriers. Co-code with EXECUTION/COMPLIANCE when accompanied by discussion about how the barrier actually led to missed doses or removal of the ring. |

Abbreviations: KC = key code (evaluated in tests of intercoder reliability); IPV: intimate partner violence; SMS: sort message service;

**Supplemental Table 3: Ethics committees approving the REACH trial**

| **Site** | **Committee(s)** |
| --- | --- |
| Kampala | Joint Clinical Research Council, Uganda National Council for Science and Technology (HS2314)  Johns Hopkins Medicine Institutional Review Boards (JHMIRB IRB00154539). |
| Harare | Joint Research Ethics Committee for The University of Zimbabwe College of Health Sciences and The Parirenyatwa Group of Hospitals (JREC/129/17)  Medical Research Council of Zimbabwe (MRCZ/A/2189) |
| Cape Town | University of Cape Town Human Research Ethics Committee (292/2017) |
| Johannesburg | University of the Witwatersrand Human Research Ethics Committee (170403B). |

**Supplemental Table 4: Additional adherence strategies selected at each visit, overall and by product type.**

|  | **Total  (n=3875)** | **Ring (n=2152)** | **Oral PrEP  (n=1723)** |
| --- | --- | --- | --- |
| In-person support club (preferred)^*^ | 42.7% | 42.8% | 42.5% |
| In-person support club (available)* | 22.9% | 22.8% | 23.0% |
| Online support club | 29.2% | 27.8% | 30.9% |
| Weekly call | 36.8% | 38.2% | 35.1% |
| Weekly SMS | 21.7% | 20.9% | 22.7% |
| Daily SMS | 25.7% | 22.2% | 30.0% |
| Additional counseling | 26.1% | 24.6% | 27.9% |
| Carrying case | 17.5% | 13.6% | 22.3% |
| Peer buddy | 4.1% | 4.0% | 4.2% |
| Other** | 13.4% | 15.1% | 11.2% |

* Because in person support clubs were not available at times due to COVID restrictions, we show the proportion of visits at which this option was selected (first row) and received (second row) separately. This distinction does not apply to the other options because they were available consistently throughout the study.

** “Other” methods selected were predominantly phone calls at less-than-weekly frequencies.

**Supplemental Figure 1. Topics discussed during counseling sessions
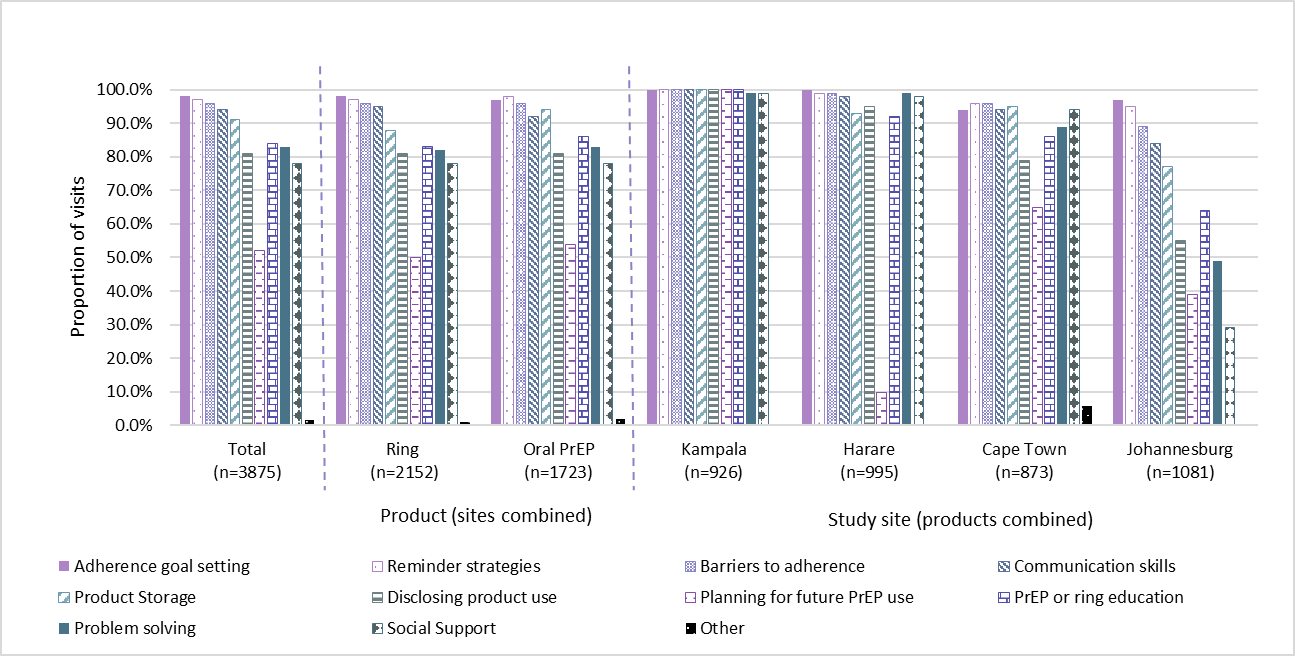
**

**Supplemental Figure 2: Percent of visits with drug level feedback results available, by site and calendar month**


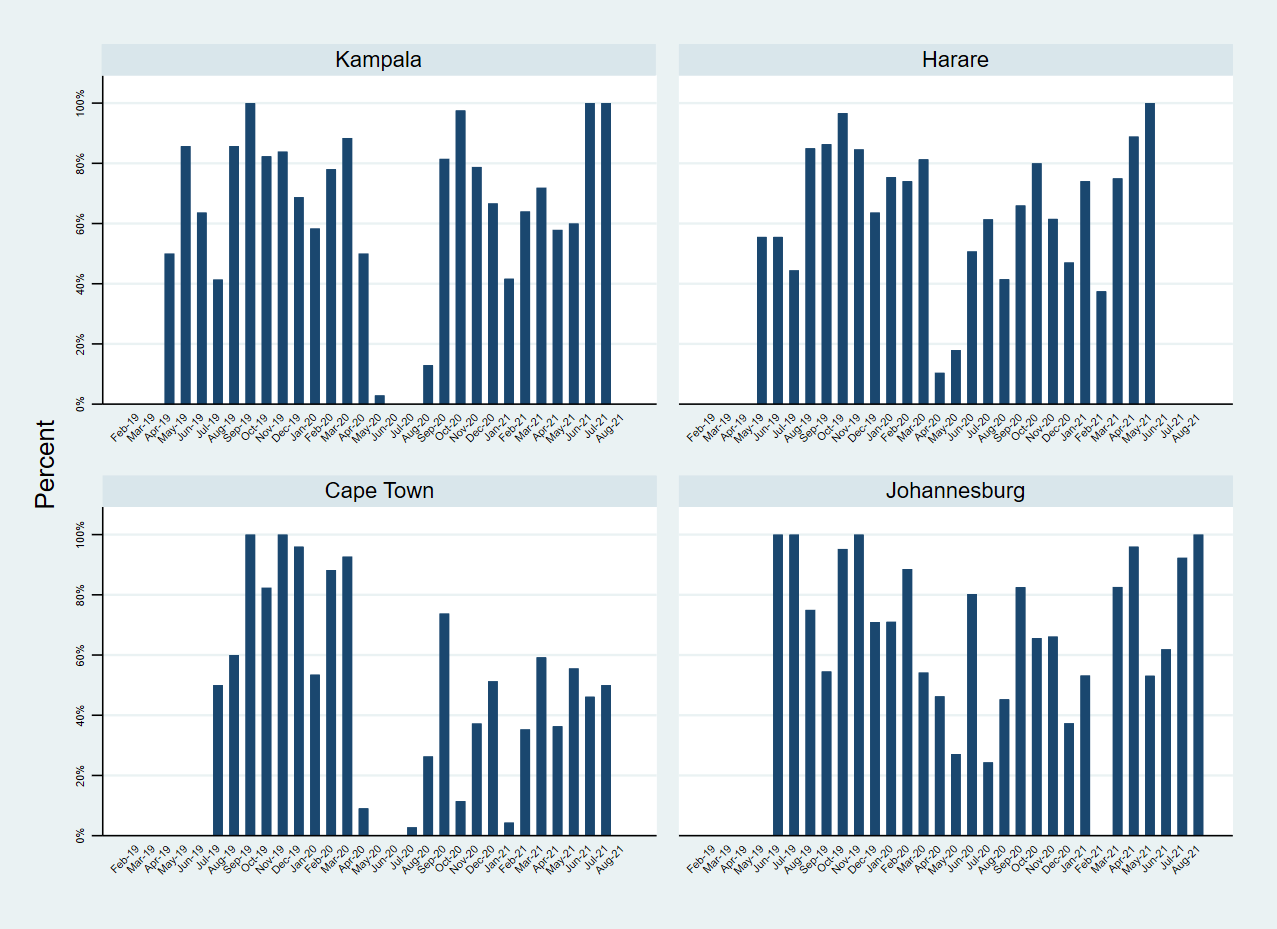

Supplement: Supplementary file 1 — Table S1: Changes to REACH adherence support activities due to the COVID‐19 pandemic. Table S2: Definitions for codes used in analysis for this manuscript. Table S3: Ethics committees approving the REACH trial. Table S4: Additional adherence strategies selected at each visit, overall and by product type. Figure S1. Topics discussed during counseling sessions. Figure S2: Percent of visits with drug level feedback results available, by site and calendar month. [file JIA2-26-e26189-s001.docx]
